# The Expected Cardiovascular Benefit of Plasma Cholesterol Lowering with or Without LDL-C Targets in Healthy Individuals at Higher Cardiovascular Risk

**DOI:** 10.5935/abc.20170089

**Published:** 2017-06

**Authors:** Fernando Henpin Yue Cesena, Antonio Gabriele Laurinavicius, Viviane A. Valente, Raquel D. Conceição, Raul D. Santos, Marcio S. Bittencourt

**Affiliations:** 1 Hospital Israelita Albert Einstein, São Paulo, SP - Brazil; 2 Instituto do Coração (InCor) - Faculdade de Medicina da Universidade de São Paulo, São Paulo, SP - Brazil; 3 Hospital Universitário da Universidade de São Paulo; São Paulo, SP - Brazil

**Keywords:** Cholesterol, HDL / blood, Cholesterol, LDL / blood, Hypercholesterolemia / blood, Risk Factors, Coronary Artery Disease

## Abstract

**Background:**

There is controversy whether management of blood cholesterol should be based or not on LDL-cholesterol (LDL-c) target concentrations.

**Objectives:**

To compare the estimated impact of different lipid-lowering strategies, based or not on LDL-c targets, on the risk of major cardiovascular events in a population with higher cardiovascular risk.

**Methods:**

We included consecutive individuals undergoing a routine health screening in a single center who had a 10-year risk for atherosclerotic cardiovascular disease (ASCVD) ≥ 7.5% (pooled cohort equations, ACC/AHA, 2013). For each individual, we simulated two strategies based on LDL-c target (≤ 100 mg/dL [S_target-100_] or ≤ 70 mg/dL [S_target-70_]) and two strategies based on percent LDL-c reduction (30% [S_30%_] or 50% [S_50%_]).

**Results:**

In 1,897 subjects (57 ± 7 years, 96% men, 10-year ASCVD risk 13.7 ± 7.1%), LDL-c would be lowered from 141 ± 33 mg/dL to 99 ± 23 mg/dL in S_30%_, 71 ± 16 mg/dL in S_50%_, 98 ± 9 mg/dL in S_target-100_, and 70 ± 2 mg/dL in S_target-70_. Ten-year ASCVD risk would be reduced to 8.8 ± 4.8% in S_50%_ and 8.9 ± 5.2 in S_target-70_. The number of major cardiovascular events prevented in 10 years per 1,000 individuals would be 32 in S_30%_, 31 in S_target-100_, 49 in S_50%_, and 48 in S_target-70_. Compared with S_target-70_, S_50%_ would prevent more events in the lower LDL-c tertile and fewer events in the higher LDL-c tertile.

**Conclusions:**

The more aggressive lipid-lowering approaches simulated in this study, based on LDL-c target or percent reduction, may potentially prevent approximately 50% more hard cardiovascular events in the population compared with the less intensive treatments. Baseline LDL-c determines which strategy (based or not on LDL-c target) is more appropriate at the individual level.

## Introduction

Lowering low-density lipoprotein-cholesterol (LDL-c) levels is a well-established way to reduce the risk of cardiovascular events^1^ and more aggressive LDL-c lowering strategies are recommended for subjects at higher risk.^2-7^ However, risk stratification, as well as recommendations for the management of blood cholesterol, differs across different guidelines.^2-7^

The latest documents from the European Society of Cardiology (ESC),^3^ European Atherosclerosis Society (EAS),^4^ National Lipid Association (NLA),^5^ Canadian Cardiovascular Society,^6^ and the Atherosclerosis Department of the Brazilian Society of Cardiology^7^ maintain the long-standing principle of establishing LDL-c target concentrations according to the absolute risk of cardiovascular events.

In 2013, however, the American College of Cardiology (ACC)/American Heart Association (AHA) cholesterol management guidelines changed this concept, abolishing the historical LDL-c targets and recommending statin prescription, at moderate or high intensity, according to the predicted absolute risk of events.^2^

Whether we should pursue LDL-c target levels or prescribe fixed-dose statins aiming at a percent LDL-c reduction is a subject of debate. Moreover, there is no consensus on how aggressive the lipid-lowering strategies should be at different risk levels, and the incremental benefit of more aggressive therapies should be counterbalanced by higher risk of adverse events and higher costs.

In order to address these issues, the purpose of this study was to compare the estimated impact of different cholesterol-lowering strategies on the risk of major cardiovascular events in healthy subjects considered to be at higher cardiovascular risk. Specifically, we simulated cholesterol-lowering approaches at different intensities and based on LDL-c target or fixed percent reduction.

## Methods

### Subjects and estimation of cardiovascular risk

Participants were selected from a large database of individuals undergoing a routine health screening at the Preventive Medicine Center of the Hospital Israelita Albert Einstein, São Paulo, Brazil, from January 2006 to June 2013. Data were prospectively collected from consecutive predominantly healthy individuals who underwent interview with a clinician, physical examination, treadmill stress test, and blood collection, among several procedures, as described elsewhere.^8^ History of cardiovascular events and current use of medication were verified. Fasting blood glucose and lipids were performed, among other tests. LDL-c was calculated using the Friedewald equation,^9^ except for the cases in which the triglyceride level was higher than 400 mg/dL, when LDL-c was measured by a direct method.

We included subjects with a calculated 10-year risk for atherosclerotic cardiovascular disease (ASCVD) ≥ 7.5%, according to the 2013 ACC/AHA risk calculator derived from the pooled cohort equations.^10^ This quantitative risk assessment method predicts the 10-year risk of developing a first cardiovascular event, defined as nonfatal myocardial infarction, coronary heart disease death, or fatal or nonfatal stroke, among people without cardiovascular disease.^10^

Exclusion criteria were as follows:

- individuals in secondary prevention, defined as history of clinical ASCVD, subclinical ASCVD deemed significant by the assistant physician, or aortic aneurysm;
- current use of lipid-lowering medication;
- presence of variable(s) out of the recommended range to use the pooled cohort equations: age < 40 years or > 79 years, total cholesterol < 130 mg/dL (3.4 mmol/L) or > 320 mg/dL (8.3 mmol/L), high-density lipoprotein-cholesterol (HDL-c) < 20 mg/dL (0.5 mmol/L) or > 100 mg/dL (2.6 mmol/L), or systolic blood pressure < 90 mmHg or >200 mmHg;
- missing data not allowing calculations needed for risk estimation or predicted cardiovascular benefit.

The study was approved by the Research Ethics Committee of the Hospital Israelita Albert Einstein, São Paulo, Brazil (CAAE: 53641916.9.0000.0071).

### Simulated strategies and assumptions

For every subject, two strategies based on LDL-c target (S_target-100_ and S_target-70_) and two based on fixed percent LDL-c reduction (S_30%_ and S_50%_) were simulated. Table 1 depicts the simulated treatments and the expected final LDL-c after the adoption of each strategy. In the strategies with LDL-c target, to reflect the common clinical practice, it was assumed that medication would be prescribed only if LDL-c was at least 20% higher than the target, and drug therapy would reduce LDL-c by at least 30%. In the strategies based on percent reduction, medication would be prescribed only if baseline LDL-c was ≥ 70 mg/dL (1.8 mmol/L), as recommended by the 2013 ACC/AHA guideline.^2^

**Table 1:** Simulated treatments and the expected final LDL-c, according to strategies and baseline LDL-c

Strategy	Baseline LDL-c	Treatment	Final LDL-c
S_30%_	< 70 mg/dL (1.8 mmol/L)	None	= baseline LDL-c
	≥ 70 mg/dL	30% LDL-c reduction	= baseline LDL-c – 30%
S_50%_	< 70 mg/dL (1.8 mmol/L)	None	= baseline LDL-c
	≥ 70 mg/dL	50% LDL-c reduction	= baseline LDL-c – 50%
S_target-100_	< 120 mg/dL (3.1 mmol/L)	None	= baseline LDL-c
	≥ 120 mg/dL	≥ 30% LDL-c reduction	= baseline LDL-c – 30% or 100 mg/dL (2.6 mmol/L)*
S_target-70_	< 84 mg/dL (2.2 mmol/L)	None	= baseline LDL-c
	≥84 mg/dL	≥ 30% LDL-c reduction	= baseline LDL-c – 30% or 70 mg/dL (1.8 mmol/L)*

* The lower value was considered.

### Estimation of cardiovascular risk reduction

The expected variation in LDL-c allowed us to estimate the absolute cardiovascular risk reduction for every individual in each of the simulated strategies. For these calculations, we considered a 22% relative risk reduction (risk ratio [RR] equal to 0.78) in major cardiovascular events for each 39 mg/dL (1 mmol/L) of LDL-c lowered, based on the Cholesterol Treatment Trialists’ (CTT) Collaboration meta-analysis.^1^ Accordingly, if LDL-c lowers 78 mg/dL (2 mmol/L), the relative risk reduces 39% (RR = 0.78 x 0.78 = 0.61). If LDL-c lowers 117 mg/dL (3 mmol/L), the relative risk is expected to reduce 53% (RR = 0.78 x 0.78 x 0.78 = 0.47).^1^ Therefore, the final cardiovascular risk was given by the following formula:

$$\begin{array}{c} \mathrm{final}\;\mathrm{cardiovascular}\;\mathrm{risk}=\\ \mathrm{baseline}\;\mathrm{cardiovascular}\;\mathrm{risk}\;x\;0.78^{n}, \end{array}$$

where *n* is the amount of LDL-c reduction expressed in mmol/L and the baseline cardiovascular risk is the ASCVD risk derived from the pooled cohort equations.^10^ Contemporary risk calculators and cost-effectiveness studies also use CTT results in order to estimate the benefits of lipid-lowering therapy.^11-14^

The number of events prevented in 10 years per 1,000 individuals assigned to a simulated strategy was calculated by dividing 1,000 by the number needed to treat (NNT), which was calculated directly as the reciprocal of the absolute difference between the baseline and the final cardiovascular risks. Calculations were also performed for subgroups defined by baseline LDL-c concentration.

### Statistical analyses

Categorical variables were expressed as proportions and the chi-square test was used in the comparisons. Continuous variables were assumed to have a normal distribution due to the large sample size and were expressed as means and standard deviations. The different strategies for cholesterol reduction were compared by multilevel mixed effects models with Bonferroni´s adjustment for multiple comparisons. Analyses were carried out with the use of the Stata software, version 13.0. P values < 0.05 were considered statistically significant.

## Results

### Study population

**Figure 1:**
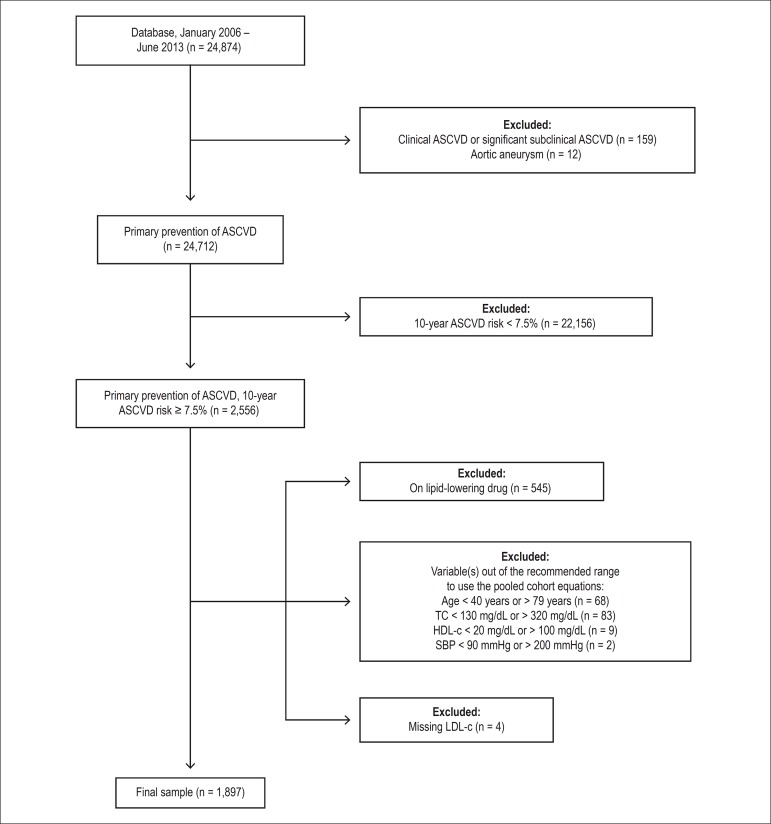
Schematic ﬂowchart depicting included and excluded subjects. ASCVD: atherosclerotic cardiovascular disease; SBP: systolic blood pressure; TC: total cholesterol.

From an initial population of 24,874 individuals, we first excluded 171 (0.7%) subjects with history of clinical ASCVD, significant subclinical ASCVD or aortic aneurysm. Among 24,712 individuals in primary prevention of ASCVD, we excluded 22,156 (89.7%) subjects with a 10-year risk for ASCVD < 7.5%. From the remaining 2,556 individuals, all of them with a 10-year risk for ASCVD ≥ 7.5%, we excluded 545 (21.3%) on current use of lipid-lowering drugs. The final study population consisted of 1,897 individuals (7.6% of the initial population, Figure 1).

### Baseline characteristics

Table 2 shows the baseline characteristics of the study subjects. The mean age was 57 ± 7 years, 96% were men, mainly white, and the mean 10-year ASCVD risk was 13.7 ± 7.1%.

**Table 2:** Baseline characteristics of participants

Characteristic	Value
Male gender	1,827 (96)
Age, years	57 ± 7
Total cholesterol, mg/dL (mmol/L)	221 ± 36 (5.7 ± 0.9)
LDL-c, mg/dL (mmol/L)	141 ± 33 (3.6 ± 0.9)
HDL-c, mg/dL (mmol/L)	43 ± 10 (1.1 ± 0.3)
Triglycerides, mg/dL (mmol/L)	190 ± 120 (2.1 ± 1.4)
Blood glucose, mg/dL (mmol/L)	102 ± 29 (5.7 ± 1.6)
Diabetes mellitus	208 (11)
Arterial hypertension	749 (40)
Smoking	590 (31)
BMI, kg/m^2^	28.4 ± 4.0
10-year ASCVD risk, %	13.7 ± 7.1

Values are expressed as mean ± SD or n (%). BMI: body mass index; ASCVD: atherosclerotic cardiovascular disease.

### Medication use

According to the LDL-c thresholds to prescribe medication showed in Table 1, the percentage of individuals receiving a lipid-lowering drug would be 99% in strategies S_30%_ and S_50%_, 74% in S_target-100_, and 96% in S_target-70_ (p < 0.001).

### LDL-c and absolute cardiovascular risk predicted reductions

**Figure 2:**
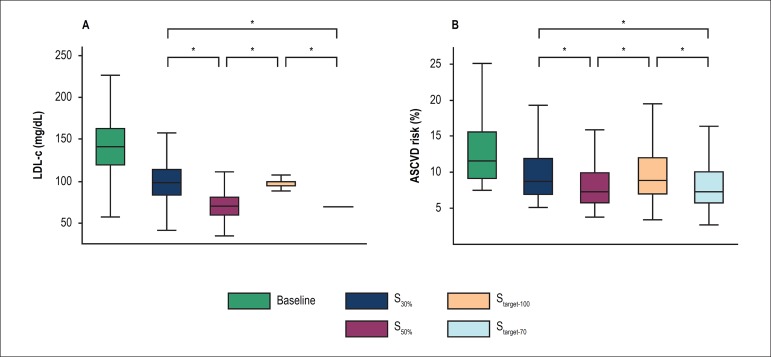
Distribution of LDL-c (A) and the 10-year risk for atherosclerotic cardiovascular disease (B) at baseline and according to the simulated strategy. ASCVD: atherosclerotic cardiovascular disease. * p <0.001.

The mean LDL-c achieved in the population would be significantly lower if participants were subjected to any of the more aggressive strategies (S_50%_ or S_target-70_), compared with the less intensive approaches (S_30%_ and S_target-100_, Figure 2A). The adoption of S_50%_ and S_target-70_ would result in numerically comparable mean LDL-c in the population (71 ± 16 mg/dL [1.8 ± 0.4 mmol/L] and 70 ± 2 mg/dL [1.8 ± 0.1 mmol/L], respectively, p = 0.039). Also, the final mean LDL-c in the population would be comparable in S_30%_ and S_target-100_ (99 ± 23 mg/dL [2.6 ± 0.6 mmol/L] and 98 ± 9 mg/dL [2.5 ± 0.2 mmol/L], respectively, p = 0.171). Of note, the distribution pattern of LDL-c in the population would be very different according to the strategy, with a wider distribution in the approaches based on percent reduction, compared with the modalities based on target concentration (Figure 2A).

In parallel to LDL-c reduction, S_50%_ and S_target-70_ would similarly decrease the average population cardiovascular risk (to 8.8 ± 4.8% and 8.9 ± 5.2%, respectively, p = 1.000), whereas both S_30%_ and S_target-100_ would reduce the mean cardiovascular risk to a comparable level (10.5 ± 5.6% and 10.6 ± 6.1%, respectively, p = 0.090). The expected final cardiovascular risk in the more aggressive strategies would be significantly lower than the risk predicted in the less intensive approaches (Figure 2B).

The number of major cardiovascular events prevented in 10 years per 1,000 individuals assigned to the strategy would be 32 in S_30%_, 31 in S_target-100_, 49 in S_50%_, and 48 in S_target-70_.

Despite resulting in similar mean values of final LDL-c and cardiovascular risk, the more aggressive strategies (S_50%_and S_target-70_) would be quite different depending on the way blood cholesterol is managed in the population. Indeed, the percentage of individuals achieving LDL-c ≤ 70 mg/dL (1.8 mmol/L) would be 98% in S_target-70_, but only 49% in S_50%_ (p < 0.001). On the other hand, while 99% of the subjects would lower LDL-c by 50% in S_50%_, this proportion would be only 52% in S_target-70_ (p < 0.001).

### Influence of baseline LDL-c on the predicted absolute risk reduction

The superiority of a strategy based on LDL-c target over percent reduction, or vice-versa, is expected to be dependent on the baseline LDL-c.

**Figure 3:**
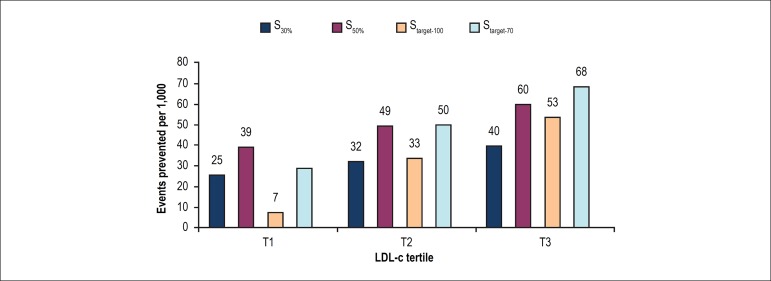
Estimated number of events prevented in 10 years per 1,000 individuals assigned to the simulated strategy, according to baseline LDL-c (lower tertile [T1]: < 129 mg/dL [3.3 mmol/L]; intermediate tertile [T2]: 129-155 mg/dL [3.3-4.0 mmol/L]; higher tertile [T3]: > 155 mg/dL [4.0 mmol/L]).

In the intermediate LDL-c tertile of our population, S_30%_ and S_target-100_ would prevent a comparable number of events, whereas S_50%_ and S_target-70_ would also be similarly effective in reducing cardiovascular events (Figure 3). The more aggressive strategies would prevent approximately 50% more hard cardiovascular events than the less intensive modalities (Figure 3).

Subjects with lower LDL-c would benefit more from S_30%_ than from S_target-100_, and more from S_50%_ than from S_target-70_. In the lower LDL-c tertile, compared with S_target-70_, S_50%_ would prevent 39% more hard cardiovascular events (Figure 3).

In individuals with higher LDL-c, S_target-100_ would prevent more events than S_30%_, and S_target-70_ would prevent 13% more cardiovascular events compared with S_50%_ (Figure 3).

## Discussion

The present study highlights relevant aspects of the management of blood cholesterol in individuals at higher cardiovascular risk:

1. the cardiovascular benefit in the population would be similar for a strategy based on a 50% LDL-c reduction or targeting LDL-c ≤ 70 mg/dL (1.8 mmol/L);
2. the cardiovascular benefit would also be similar for a strategy based on a 30% LDL-c reduction or targeting LDL-c ≤ 100 mg/dL (2.6 mmol/L);
3. LDL-c lowering strategies based on target level or percent reduction may promote similar overall cardiovascular benefit despite resulting in different LDL-c distribution patterns in the population;
4. the more aggressive modalities (based on a 50% LDL-c reduction or targeting LDL-c ≤ 70 mg/dL) would prevent approximately 50% more hard cardiovascular events compared with the less intensive treatments;
5. baseline LDL-c determines which modality of treatment (based on target concentration or percent reduction) would prevent more cardiovascular events.

Since the publication of the 2013 ACC/AHA cholesterol management guidelines^2^ recommending a switch from a treat-to-target approach to a statin dose-based strategy, intense debate has taken place both inside and outside the USA.^15-17^ In the absence of randomized clinical trials directly comparing the outcome of different strategies, with or without LDL-c target concentrations, simulations may be of value to provide information that may help guide therapy and develop guidelines.

When recommending LDL-c target levels, guidelines differ on the goal for individuals in primary prevention at higher cardiovascular risk (< 70 mg/dL [1.8 mmol/L),^3,4,7^ < 77 mg/dL [2.0 mmol/L)^6^ or < 100 mg/dL [2.6 mmol/L]^5^). An aggressive LDL-c target (< 70 mg/dL [1.8 mmol/L]) for these patients is supported by the CTT meta-analysis,^1^ as well as by extrapolation of subanalyses of randomized clinical trials in patients with cardiovascular disease.^18,19^ Accordingly, our study, which is based on assumptions derived from the CTT study,^1^ showed a robust difference between achieving LDL-c ≤ 70 mg/dL or ≤ 100 mg/dL. This finding, however, contrasts with a recent population-based study that reported no additional benefit by achieving LDL-c 70 mg/dL or less in individuals with stable ischemic heart disease taking statins.^20^

One of the main criticisms of the abolishment of LDL-c target levels is the possibility of undertreating individuals with higher baseline LDL-c. Indeed, subjects with LDL-c > 140 mg/dL will not achieve 70 mg/dL even if they reduce LDL-c by 50%, approximately the expected mean reduction with high-intensity statin. In our simulations, this phenomenon was not negligible, as more than half of the study population simulated to a 50% LDL-c reduction would not attain LDL-c ≤ 70 mg/dL. This result compares with those of a meta-analysis of statin trials showing that more than 40% of subjects assigned to high-dose statin therapy did not reach LDL-c target < 70 mg/dL.^21^ In this regard, it is noteworthy that the expert consensus published by the ACC in 2016 states that a non-statin drug (ezetimibe) may be considered for primary prevention patients with 10-year ASCVD risk ≥ 7.5% and high-risk markers who have not achieved LDL-c < 100 mg/dL on maximally tolerated statin therapy.^22^

On the other hand, under an LDL-c target-based strategy, many individuals with baseline LDL-c in the lower range would not need high-dose statins to reach the lipid goal. These individuals may also be considered undertreated, since high-dose statins would promote higher absolute LDL-c lowering and higher cardiovascular risk reduction. Our data demonstrate that this situation should not be underestimated. Indeed, in perfect agreement with our results, the recently published European guidelines recommend, for very high-risk patients, an LDL-c goal < 70 mg/dL (1.8 mmol/L) or a reduction by at least 50% if LDL-c is between 70 and 135 mg/dL (1.8 and 3.5 mmol/L).^3,4^

The importance of a percent LDL-c reduction is also supported by a recent publication by Bangalore et al.^23^ In a large cohort of patients included in randomized trials, the authors reported that the percent LDL-c reduction added incremental prognostic value over statin dose and attained LDL-c levels, but achieved LDL-c did not provide incremental prognostic value over statin dose and percent LDL-c reduction.^23^

Therefore, the present study supports treating those individuals with relatively higher LDL-c plasma concentration to aggressive LDL-c target levels and prescribing high-dose statin, aiming for a great percent LDL-c reduction, for those with relatively lower LDL-c level. Our data suggest that the debate should shift from the “with or without target” issue to a broader discussion about how to customize the management of blood cholesterol in order to minimize the impact of cardiovascular diseases in the population.

### Limitations

This study has several limitations inherent to simulation analyses. We had to make some assumptions arbitrarily and we cannot exclude the possibility of different results if other assumptions were used. We also simulated 30% and 50% LDL-c reductions as strategies roughly representative of moderate- and high-intensity statin therapies, respectively. It is widely known, however, that there is a substantial interindividual variability in the response to statin therapy.^21,24,25^

Our study population was almost exclusively male because of intrinsic characteristics of the preventive service where data were collected, which is attended predominantly by businessmen. Different results may be seen in populations with a more balanced male/female ratio. Ethnic issues also have to be considered, since we studied an almost exclusively white population. Importantly, we predict that results may significantly vary according to mean LDL-c in the population. Therefore, we cannot extrapolate our findings to other communities.

## Conclusions

In a simulation study based on real-world individuals considered to be at higher risk for cardiovascular events, we observed that LDL-c lowering strategies based on target level or percent reduction may promote similar overall cardiovascular benefit, despite resulting in different distribution patterns of LDL-c in the population. Importantly, both aggressive approaches simulated (S_50%_ and S_target-70_) may potentially prevent approximately 50% more hard cardiovascular events in the population compared with the less intensive treatments (S_30%_ and S_target-100_).

However, these strategies may be very different at the individual level depending on the baseline LDL-c. An aggressive target-based strategy is the best option when LDL-c is relatively high, while percent LDL-c reduction is superior when LDL-c is relatively low.
